# Fluorescence microscopy data on expression of Paired Box Transcription Factor 7 in skeletal muscle of APOBEC2 knockout mice

**DOI:** 10.1016/j.dib.2018.02.063

**Published:** 2018-02-27

**Authors:** Hideaki Ohtsubo, Yusuke Sato, Yuji Matsuyoshi, Takahiro Suzuki, Wataru Mizunoya, Mako Nakamura, Ryuichi Tatsumi, Yoshihide Ikeuchi

**Affiliations:** a Department of Animal and Marine Bioresource Sciences, Graduate School of Agriculture, Kyushu University, Hakozaki, Fukuoka 812-8581, Japan; b Department of Bio-Productive Science, Utsunomiya University, Utsunomiya, Tochigi 321-8505, Japan; c Department of Molecular and Developmental Biology, Kawasaki Medical School, Kurashiki, Okayama 701-0192, Japan; d Graduate School of Agriculture, Kyushu University, Hakozaki, Fukuoka 812-8581, Japan

**Keywords:** APOBEC2, Myogenic stem satellite cells, Self-renewal, Muscle, Cryo-sections, Immunofluorescence

## Abstract

The data presented in this article are related to the research articles entitled “APOBEC2 negatively regulates myoblast differentiation in muscle regeneration” and “Data supporting possible implication of APOBEC2 in self-renewal functions of myogenic stem satellite cells: toward understanding the negative regulation of myoblast differentiation” (Ohtsubo et al., 2017a, 2017b) [1,2]. This article provides *in vivo* phenotypical data to show that Paired Box Transcription Factor 7 (Pax7)-positive cell number (per myofiber) is significantly lower in APOBEC2 (a member of apoB mRNA editing enzyme, catalytic polypeptide-like family)-knockout muscle than the control wild-type tissue at the same age of 8-wk-old in mice. The emerging results support an essential role for APOBEC2 in the self-renewal functions of myogenic stem satellite cells, namely the re-establishment of quiescent status after activation and proliferation of myoblasts.

**Specifications Table**

**Table:** 

Subject area	*Biology*
More specific subject area	*Skeletal muscle biology, tissue-specific stem cell physiology*
Type of data	*Image (microscopy), graph*
How data was acquired	*Fluorescence microscopic system (Leica DMI6000B fluorescence microscope equipped with a DFC365FX digital camera and LAS AF 3.1.0 software)*
Data format	*Raw (microscopy), analyzed (Pax7-positive cell counting)*
Experimental factors	*Cryo-cross sections of tibialis anterior (TA) muscle from adult male wild type and APOBEC2-knockout (KO) mice, double-immunostained with anti-Pax7 and anti-laminin antibodies, and counted for Pax7-positive cell number per myofiber*
Experimental features	*Pax7/laminin-immunofluorescence microscopy*
Data source location	*Fukuoka, Japan*
Data accessibility	*All relevant data are within the article*

**Value of the data**

• Resident myogenic stem satellite cell population concerned here is a valuable target of research on *postnatal* muscle fiber growth, hyperplasia/hypertrophy, regeneration, fiber-type commitment, and moto-neuritogenesis.
• Molecular mechanisms for self-renewal functions of satellite cells are important research subjects and hence of value to the scientific community.
• APOBEC2 expression is predominant in skeletal and cardiac muscles and elevated exclusively at the early-differentiation phase of myoblasts in muscle regeneration; however the biological and physiological significance is still unclear (see Refs. [3], [4]).
• The particular idea of an essential role for APOBEC2 in the self-renewal functions was raised by the previous study in single myofiber culture (see Ref. [2]) and further supported by the present *in vivo* study to show that Pax7-positive satellite cell population was significantly lower in APOBEC2-KO muscle than the control at the same adult phase in mice.
• The idea extends our understanding of the previous finding that APOBEC2 negatively drives regulation of myoblast differentiation and fusion (see Ref. [1]).

## 1 Data

**Fig. 1:**
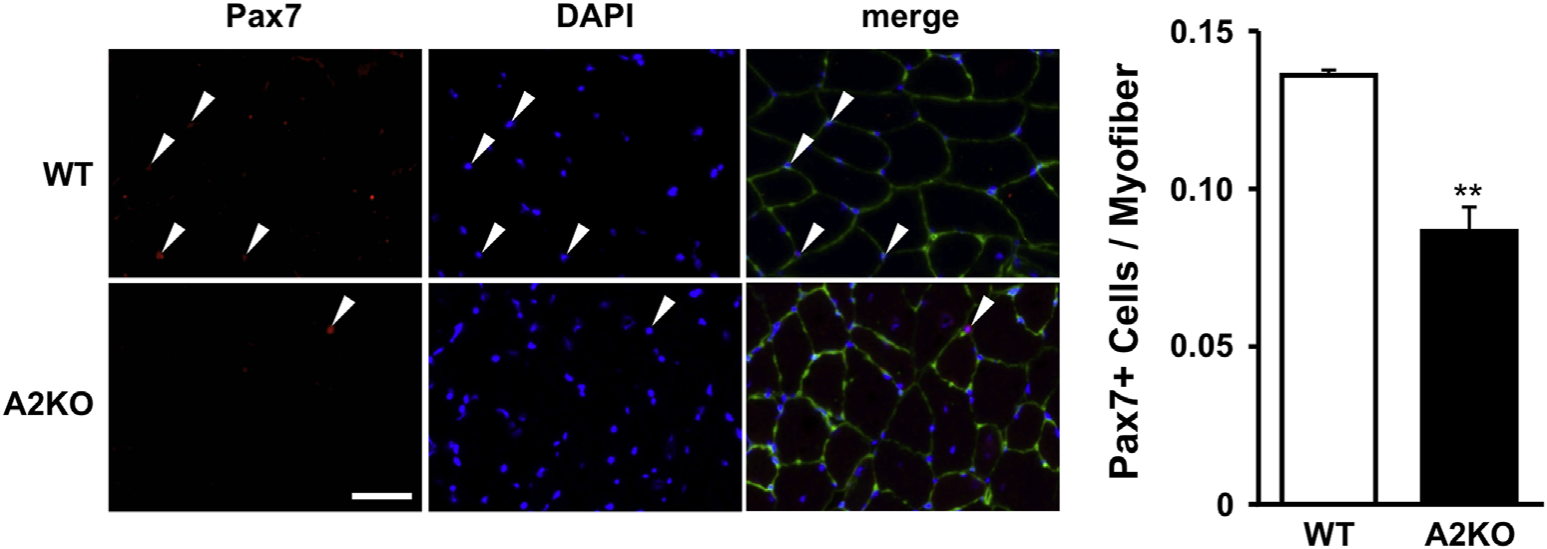
Effect of APOBEC2 deficiency on satellite cell number in skeletal muscle tissue. The left panel shows fluorescence micrographs of TA muscle cross-sections, which were double-immunostained with anti-Pax7 (*fluorescent red*; satellite cells) and anti-laminin antibodies (*green*; basal lamina) along with counter-staining with DAPI (*blue*, nuclei). Representative Pax7-positive cells were indicated by arrow heads (upper row, WT; lower row, APOBEC2-KO mice (A2KO), at 8-wks old). Scale bar = 50 μm. The bar graph shows Pax7-positive cell number per myofiber in both groups (*n* = 3 mice per group, mean ± S.E.M., ** *p* < 0.01 *vs*. WT).

We tested a hypothesis that cytidine deaminase APOBEC2 may be an important mediator in the self-renewal functions of satellite cells, namely in the re-establishment of quiescent status after activation and proliferation of myoblasts. De Luca et al. [5] reported that defect of the ability of satellite cell self-renewal led to diminished number of satellite cells in skeletal muscle tissues. Accordingly, we compared satellite cell number in TA muscle between wild-type (WT) and APOBEC2-KO mice by immunofluorescence using antibody against Pax7, a well-known reliable marker for quiescent satellite cells. In APOBEC2-KO muscle cross-sections, Pax7-positive cell number (per myofiber) was significantly lower than the control WT tissue at the same age (*p* < 0.01), supporting the hypothesis that APOBEC2 deficiency leads to defect of the self-renewal of satellite cells (Fig. 1).

## 2 Experimental design, materials and methods

### 2.1 Experimental design

To further evaluate the above particular idea of a role for APOBEC2 in the self-renewal functions, TA muscles were collected from adult male WT and APOBEC2-KO mice (8-wk-old) and assayed for the number of quiescent myogenic stem satellite cells (Pax7-positive cells) on cryo-sections by Pax7-immunofluorescence microscopy.

### 2.2 Materials and methods

#### 2.2.1 Animal care and use

APOBEC2-KO mice (C57BL/6 as the background strain) were generated by Dr. Neuberger (Medical Research Council Laboratory of Molecular Biology, United Kingdom) [6] and bred in our laboratory. Inbred C57BL/6 mice were used as WT controls. All animal experiments were conducted in strict accordance with the Guidelines for Proper Conduct of Animal Experiments published by the Science Council of Japan and ethics approvals from the Kyushu University Institutional Review Board (approval nos. 20-12, 23-62, A22-218, A24-75, A26-078, and A28-090).

#### 2.2.2 Double-immunofluorescence microscopy for quiescent satellite cell population analyses

Cross-sections of TA muscle from 8-wk-old adult male WT and APOBEC2-KO mice were fixed by incubation in phosphate-buffered saline (PBS) at 90 °C for 10 min and blocked with normal donkey blocking solution (2% donkey normal serum, 1% bovine serum albumin, 0.1% cold fish skin gelatin, 0.1% Triton X-100, 0.05% Tween 20, 0.05% sodium azide, 0.01% avidin, and 100 mM mannose in PBS). Subsequently, sections were incubated in a mixture of monoclonal anti-Pax7 (1:50 dilution in blocking solution; obtained from the Developmental Studies Hybridoma Bank, Iowa City, IA, USA) and polyclonal anti-laminin antibodies (1:500 dilution; L9393 from Sigma-Aldrich, St. Louis, MO, USA). Alexa Fluor 594-labeled anti-mouse IgG (1:500 dilution; A21201, Invitrogen, Grand Island, NY, USA) and Alexa Fluor 488 anti-rabbit IgG secondary antibodies (1:500 dilution; A-21441, Invitrogen) were used as secondary antibodies, respectively. Sections were counter-stained with 4′,6-diamidino-2- phenylindole (DAPI; D523 purchased from DOJINDO Laboratories, Kumamoto, Japan) and observed under a Leica DMI6000B fluorescence microscope equipped with a DFC365FX digital camera and LAS AF 3.1.0 software. Pax7-positive cells on WT and APOBEC2-KO tissue sections were counted and assigned to satellite cell number per myofiber (*n* = 3 mice per group).

#### 2.2.3 Statistical analysis

Student's *t-*test was employed for statistical analysis of experimental results using Microsoft Excel X for Macintosh. Data are represented as mean ± S.E.M. for three mice per group and the level of significance was set to *p* < 0.05.

## Funding sources

This work was funded by Grant-in-Aid for Scientific Research (B) 23380159 from the Japan Society for the Promotion of Science (JSPS) (to Y. Ikeuchi). Research was also supported, in part, by Grants-in-Aid for Scientific Research (A) 16H02585 and (B) 22380145, 25292164 and 17H03908, by the Invitation Fellowship Program for Research in Japan (JSPS), and by grant funds from the Ito Foundation, the Uehara Memorial Foundation, and Graduate School of Agriculture, Kyushu University (all to R. Tatsumi). H. Ohtsubo received a scholarship from Kyushu University during the course of this research.
